# Some Alternatives? Event-Related Potential Investigation of Literal and Pragmatic Interpretations of *Some* Presented in Isolation

**DOI:** 10.3389/fpsyg.2016.01479

**Published:** 2016-09-30

**Authors:** Cécile Barbet, Guillaume Thierry

**Affiliations:** School of Psychology, Bangor UniversityBangor, UK

**Keywords:** scalar inferences, inter-individual variation, pragmatic tolerance, systemizing, event-related potentials, P3b

## Abstract

In sentence verification tasks involving under-informative statements such as *Some elephants are mammals*, some adults appear more tolerant to pragmatic violations than others. The underlying causes of such inter-individual variability remain however essentially unknown. Here, we investigated inter-individual variation in adults deriving the scalar inference “not all” triggered by the quantifier *some*. We first assessed the individual intolerance to pragmatic violations in adult participants presented with under-informative *some*-statements (e.g., *Some infants are young*). We then recorded event-related brain potentials in the same participants using an oddball paradigm where an ambiguous deviant word *some* presented in isolation had to be taken either as a match (in its literal interpretation “at least some”) or as a mismatch (in its pragmatic interpretation “some but not all”) and where an unambiguous deviant target word *all* was featured as control. Mean amplitude modulation of the classic P3b provided a measure of the ease with which participants considered *some* and *all* as deviants within each experimental block. We found that intolerance to pragmatic violations was associated with a reduction in the magnitude of the P3b effect elicited by the target *some* when it was to be considered a literal match. Furthermore, we failed to replicate a straightforward literal interpretation facilitation effect in our experiment which offers a control for task demands. We propose that the derivation of scalar inferences also relies on general, but flexible, mismatch resolution processes.

## 1. Introduction

Understanding the meaning of sentences involves two kinds of processes: (i) decoding literal meaning and (ii) deriving inferences that go beyond the literal meaning of words and clauses (*implicatures*, see e.g., Grice, 1968). For example, in:

(1) Anna: Did the children's summer camp go well?
         Bob: Some of them got stomach flu.
  - More than one child/at least some of the children got stomach flu.
  - Not all the children got stomach flu.
  - The summer camp didn't go as well as hoped (from Carston, 2004).

while the literal meaning of Bob's answer is (1-a), Anna can infer from his answer both (1-b) and (1-c). According to Gricean terminology (see e.g., Grice, 1968; Levinson, 2000), (1-b.) is a *generalized* conversational implicature because it is triggered by a specific item (*some*) and is assumed to arise generally across contexts; while (1-c) is a *particularized* conversational implicature because it crucially depends on the context. Indeed if Anna's question had been “Were all children able to sit their exams?” for example, the inference (1-c) would not arise; whereas if Anna's question concerned the exams rather than the summer camp (1-b) would still hold.

Generalized inferences such as (1-b) are called *scalar inferences* (hereafter SIs) because they are triggered by linguistic expressions which have stronger competitors on scales of informativeness (see Horn, 1972, 1989). For instance, in (1), *some* contrasts with *all* and thus can trigger the SI “not all.” Other examples of lexical scales are 〈always, sometimes〉, 〈and, or〉, 〈finish, start〉, 〈impossible, difficult〉 (see e.g., van Tiel et al., 2016).

In Gricean pragmatics, drawing an SI requires at least two steps (see e.g., Katsos and Bishop, 2011; Bott et al., 2012; Breheny et al., 2013). First, the hearer determines whether the speaker could have made a more informative (i.e., stronger) statement; then she negates the alternative statement because she assumes that the speaker would have chosen the stronger statement if it had been true. In certain semantic contexts, e.g., antecedents of conditionals (see “downward entailing” contexts in Chierchia, 2004), such as:

(2) If some of the students fail the test, their teacher will be disappointed (Katsos et al., 2005, p. 1108).

it is not expected that the SI will be drawn since an alternative sentence with a stronger term would be informatively weaker (see e.g., Hartshorne et al., 2015; Politzer-Ahles and Gwilliams, 2015). Moreover, depending on context, the hearer may or may not negate the alternative statement when it is stronger according to assumed speaker knowledge in a second step, also called the *epistemic* step (see e.g., Breheny et al., 2013). Therefore, we can expect a hearer of:

(3) At my client's request, I skimmed the investment report. Some of the real estate investments lost money (Bergen and Grodner, 2012).

to stick to the literal meaning of *some* because the speaker is assumed to have insufficient knowledge of the situation to warrant the use of the stronger alternative *all*. On the contrary, a hearer of:

(4) At my client's request, I meticulously compiled the investment report. Some of the real estate investments lost money (Bergen and Grodner, 2012).

should draw the SI, since the speaker can be inferred to have exhaustive information about the case.

Scalar inferences have become the test case in experimental pragmatics for more than a decade in the debate opposing tenets of possible automatic inference derivation (the “default models,” inspired by Levinson, 2000; Chierchia, 2004) and tenets of context-dependency, arguing that generalized implicatures do not exist (the “context-driven models,” inspired by Carston, 1995; Sperber and Wilson, 1995). SI context-sensitivity has been shown in a number of experimental studies (see e.g., Breheny et al., 2006; Bergen and Grodner, 2012; Politzer-Ahles and Fiorentino, 2013; Hartshorne et al., 2015), but expected delays or processing costs associated with their derivation have not always been observed (see e.g., Grodner et al., 2010; Breheny et al., 2013; Politzer-Ahles and Fiorentino, 2013; Degen and Tanenhaus, 2015; Hartshorne et al., 2015). Consequently, a constraint-based formulation of context-driven models has been proposed according to which SI derivation can appear default-like when enough linguistic and contextual cues are present and reduce processing delay or cost (see Grodner et al., 2010; Degen and Tanenhaus, 2011, 2015).

However, models in experimental pragmatics have paid less attention to inter-individual variation (but see Feeney et al., 2004; Nieuwland et al., 2010; Antoniou and Katsos, 2011; Heyman and Schaeken, 2015; Zhao et al., 2015). In sentence verification paradigms involving under-informative sentences such as:

(5) Some elephants are mammals (Bott and Noveck, 2004).

some adult participants tend to consistently accept such under-informative statements that are literally true but pragmatically infelicitous (not just *some*, but *all* elephants are mammals) while other tend to consistently reject them (see e.g., Noveck and Posada, 2003; Feeney et al., 2004; Antoniou and Katsos, 2011; Hunt et al., 2013). This led to a distinction between “literal” (or “logical”) and “pragmatic” responders. Moreover, because rejecting under-informative statements took more time than accepting them, it was assumed that literal responses did not require computation of the SI. However, in Feeney et al. (2004) or Antoniou and Katsos (2011), participants needed more time to accept under-informative *some*-statements than informative *some*-statements such as:

(6) Some men have beards (Feeney et al., 2004).

Such a result is not expected if one assumes that the SI is not computed at all in the case of literal responses to under-informative statements. Therefore, Antoniou and Katsos (2011) proposed that all adult participants are sensitive to violations of informativeness and thus, that all consider whether or not a more informative statement with a stronger expression could have been used. Katsos and Bishop (2011, p. 77) stressed that responses to under-informative statements in forced-choice paradigms may also reflect a metalinguistic decision to “reject the utterance as worse than optimal or to accept it as better than false.” That being said, a consistently literal vs. pragmatic response pattern could also reflect a desire of within-task consistency on the part of participants. Indeed, since the test sentences can be interpreted as either true or false and the choice is forced, participants may initially randomly opt for true or false and then stick to their initial choice in order to maintain idiosyncratic consistency (see also Tavano and Kaiser, 2010).

Since they are able to fully derive SIs, one wonders why some adult participants accept under-informative statements at all. If a literal or pragmatic response pattern^1^ is not essentially accounted for by different strategic and/or metalinguistic processes, one hypothesis is that participants who are led to interpret *some* literally or pragmatically might experience some difficulty shifting from one to the other interpretation. Here we sought to obtain an independent, quantitative, and objective measure of pragmatic or literal functioning in participants construed as pragmatic or literal on the basis of their performance in a sentence evaluation task, using event-related potentials (ERPs).

Previous ERP studies using under-informative segments have provided some evidence that pragmatically skilled participants (as indexed by sub-scale(s) of the Autism-Spectrum Quotient questionnaire) are more sensitive to violations of informativeness than their less pragmatically skilled peers (Nieuwland et al., 2010, N400 study; Zhao et al., 2015, MMN study). To our knowledge, no study to date has investigated inter-individual variation in participants led to behave pragmatically or literally. In the present study, we invited participants to consider *some* in its literal or pragmatic sense via direct instruction (see also Bott and Noveck, 2004; Bott et al., 2012; Tomlinson et al., 2013) rather than constrain the interpretation of *some* based on cues derived from the linguistic context. This is because conditions are never fully comparable even when considering elegantly designed studies in which context control was maximal. For instance, in Politzer-Ahles and Fiorentino (2013) and Politzer-Ahles and Gwilliams (2015), *any* vs. *all* were used in the contexts preceding *some*. However, *any* and *some* are more strongly associated than *all* and *some* (see e.g., the Edinburgh Associative Thesaurus Kiss et al., 1973) leading to the *any*-contexts possibly being more predictive of *some* than the *all*-contexts. Moreover, in order to focus the quantitative ERP measure on the critical word *some*, we resorted to present it in isolation. Furthermore, we used a pragmatically unambiguous stimulus *all* as control whereas the ambiguous stimulus *some* was to be considered in its literal (*at-least-some*) or its pragmatic (*some-but-not-all*) sense depending on instruction given at the onset of each experimental block.

The P300 wave (or P3, see e.g., Luck, 2005; Polich, 2007) which is a positive-going ERP deflection peaking between 250 and 500 *ms* (or even later depending on experimental parameters, see e.g., Picton, 1992; Polich, 2007) is commonly elicited by deviant stimuli in *oddball* paradigms. In an oddball paradigm, stimuli of lower relative probability called *deviants* are presented within pseudo-randomly structured streams of higher relative probability stimuli called *standards*. Participants are usually asked to detect a particular type of deviant stimulus called *target*. Target detection is classically associated with an instance of the P300 –the P3b– maximal over parietal areas of the scalp, commonly accepted as an index of conscious target detection and working memory updating (see e.g., Donchin, 1981; Polich, 2007). In the present study, we used the P3b as an index of target-likeness for the words *all* and *some*, the latter depending on the instructions provided to the participant at the beginning of each block. In other words, the P3b provided a quantitative, objective, and context-free measure of the ease with which participants implemented the pragmatic or literal interpretation of *some*, when instructed to do so. Thus, we expected the amplitude of the P3b to increase proportionally to the target-likeness of *some* under different instructions, that is, it would measure the effectiveness with which participants acted pragmatically or literally. Furthermore, the absence of a “pragmatic N400” in the study by Nieuwland et al. (2010) might relate to strategic effects: Participants who show no significant “pragmatic N400” might have rapidly become aware that half of the sentences starting with *some* were strange and made sense only after the comma. This might have reduced N400 amplitude because under-informativeness gradually became more expected with time. The P3b is thus arguably a better index since its amplitude does not decrease with time.

In the ERP experiment, participants were presented with single quantifiers or numerals (ALL, SOME, ONE, TWO, NONE, or THREE), printed in white and green letters on a black background. Sometimes the number of green letters within a word stimulus matched its meaning (e.g., ALL printed with all of its three letters in green) and sometimes there was a mismatch (e.g., ALL printed with only some of its letters in green). In half of the blocks, participants were instructed to consider the word SOME printed with all its letters in green (ambiguous-SOME) as a mismatch (because not *some*, but *all* letters are green) and in the other blocks as a match (because if *all* of its letters are green, then *some* of them necessarily are). The unambiguous match or mismatch stimulus ALL served as control and we manipulated the ratio of match and mismatch stimuli so as to obtain an oddball distribution prone to eliciting a P3b. Experimental blocks were of two types, based on whether participants had to detect match words within a stream of mismatch ones (*match target* blocks) or mismatch words within a stream of match ones (*mismatch target* blocks). The full design of the ERP experiment is depicted in Table 2 in Section 2.2.2.

Before being engaged in the oddball paradigm, participants completed a questionnaire assessing their pragmatic tolerance based on acceptability judgements (how strongly they agree or disagree with under-informative statements such as “Some circles are round”). The questionnaire also assessed Autism-Spectrum Quotient, Empathy Quotient, Interpersonal Reactivity Index and Systemizing Quotient in order to shed light on the personality traits or cognitive style that could account for tolerance or intolerance to pragmatic violations.

From a behavioral point of view, in the ERP experiment, we expected a general facilitation effect when *some* was to be taken in its literal interpretation as observed in a number of previous studies (see e.g., Noveck and Posada, 2003; Bott and Noveck, 2004; De Neys and Schaeken, 2007; Chevallier et al., 2008; Bott et al., 2012). We did not have any prediction regarding possible relationships between the participants' pragmatic tolerance as measured by the questionnaire and behavioral data. In contrast, we expected to find a relationship between pragmatic tolerance and the magnitude of the P3b effect elicited by the critical ambiguous stimulus SOME, depending on whether it was to be considered literal or pragmatic. More specifically, if SOME was to be taken literally, we expected the magnitude of the P3b effect to be particularly pronounced for literal responders and less so for pragmatic ones, whereas the reverse pattern should be observed when SOME was to be taken pragmatically.

## 2. Methods

### 2.1. Participants

Fifty-two native speakers of English (33 females; mean age = 21.2, *SD* = 4.7) gave written consent to take part in the experiment approved by the Ethics Committee of Bangor University, United Kingdom. All were students from the School of Psychology and were given course credits for their participation. All had normal or corrected-to-normal vision. No EEG data was recorded for one participant due to a technical fault and the data of 12 participants had to be dismissed due to excessive artifacts (see Section 2.4 for details). Therefore, statistical analyses of ERP results are based on 39 individual datasets, and behavioral results (reaction times and accuracy) on 38 individual datasets because one behavioral dataset was missing due to a technical error.

### 2.2. Materials

#### 2.2.1. Questionnaire

The questionnaire comprised the 50 statements of the Autism-Spectrum Quotient questionnaire (henceforth AQ), the 60 statements of the Empathy Quotient questionnaire (EQ), the 75 statements of the Systemizing Quotient-Revised questionnaire (SQ-R), the 28 statements of the Interpersonal Reactivity Index (IRI) and 40 *all*- or *some*-statements.

The AQ, EQ, IRI, and SQ-R are self-report questionnaires for use with adults with normal intelligence.

The AQ measures the degree to which a person presents the traits associated with the autistic spectrum (Baron-Cohen et al., 2001). It includes 10 statements from the 5 following sub-scales: *social skill, attention switching, attention to detail, imagination* and *communication*. Given the topic of this study and the results obtained by Nieuwland et al. (2010), we focused on the *communication* sub-scale of the AQ.

The EQ measures individual differences in empathy (Baron-Cohen and Wheelwright, 2004). It comprises 40 empathy items and 20 filler items. The EQ does not distinguish affective from cognitive empathy; nevertheless, SI derivation does not appear related to affective empathy but rather to some form of mind-reading akin to cognitive empathy (see e.g., Pijnacker et al., 2009). Therefore, we also included the IRI, which is another instrument developed in order to measure individual differences in empathy, assessing 4 different areas (with 7 items per area): *empathic concern, personal distress, fantasy*, and *perspective-taking* (Davis, 1980, 1983). The first two areas concern affective empathy whilst the two others relate to cognitive empathy. Since step 2 of SI derivation entails evaluating the epistemic state of the speaker, we focused on the *perspective-taking* sub-scale.

Finally, the SQ-R measures individual differences in systemizing, that is the ability to analyse systems, extract rules, and predict system outputs (Wheelwright et al., 2006; Baron-Cohen, 2008, 2009, 2011). We included this measure to test the hypothesis that high systemizing ability can help reject under-informative statements. This idea arose from our reading of studies investigating high-functioning individuals with autism and Asperger's syndrome (e.g., Pijnacker et al., 2009), individuals who are very good at systemizing (see e.g., Wheelwright et al., 2006). Despite their associated high score on the AQ *communication* sub-scale, they appear to derive SIs as often as control participants, although the high AQ *communication* score predict poorer pragmatic skills (Pijnacker et al., 2009; Chevallier et al., 2010; see also Nieuwland et al., 2010, p. 343).

The 40 *all*- or *some*-statements were either true or false but, in the case of *some*-statements, possibly under-informative (i.e., logically true but pragmatically infelicitous). There were 10 such *some*-statements, 10 true and 10 false control *all*-statements, and 5 true and 5 false control *some*-statements. We computed a Pragmatism score on the basis of the responses to the 10 under-informative *some*-statements. As in Noveck (2001) and Feeney et al. (2004), participants were randomly assigned to one of two lists in order to minimize item-driven effects^2^ (see Table 1 for examples of statements and Table A1 in Appendix A for the full lists). Some of the statements were taken from previous studies (Noveck and Posada, 2003; Feeney et al., 2004; Banga et al., 2009; Nieuwland et al., 2010).

**Table 1:** **Examples of *all*- and *some*-statements used in the questionnaire**.

**Condition**	**Example**
Test existentials	*Some circles are round*
True universals	*All infants are young*
False universals	*All animals are black*
True existentials	*Some children are blonde*
False existentials	*Some books are good to eat*

Participants were asked to choose between “strongly agree,” “slightly agree,” “slightly disagree,” or “strongly disagree” in response to each statement (we adapted the 5-level scale of the IRI to fit this scale used in the AQ, EQ, and SQ-R). The *all*- and *some*-statements were mixed with AQ, EQ, SQ-R, and IRI statements so as to reduce consistency within-task effects (see Section 1, see also Feeney et al., 2004, p. 127). We thus used the same 4-level scale for the *all*- and *some*-statements as for the AQ, EQ, SQ-R, and IRI statements. Furthermore, we assumed that using a 4-level scale for critical under-informative *some*-statements might increase sensitivity as compared to a binary forced-choice (true/false). “Strongly agree” answers to these statements were scored 0, “slightly agree” answers were scored 1, “slightly disagree” answers were scored 2 and “strongly disagree” answers were scored 3. Therefore, the range of Pragmatism score was 0–30, low scores indicating tolerance to pragmatic violations and high scores indicating intolerance to such violations.

#### 2.2.2. ERP experiment

**Figure A1:**
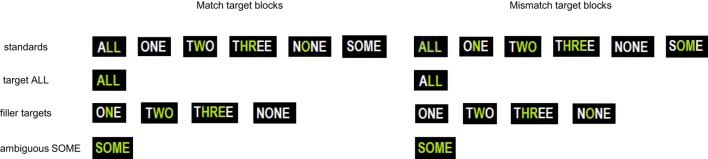
**Material used in the ERP experiment (see Section 2.2.2)**.

Using the words *all, some, none, one, two*, and *three* we constructed 12 stimuli using white and green letters, the number of green letters being consistent or not with the meaning of the word (see Figure A1 in Appendix B). Using a bold typeface to represent letters presented in green and a light typeface to represent letters presented in white, match stimuli were: **ALL**, S**OM**E, NONE, O**N**E, T**WO**, T**HRE**E, and mismatches were: A**LL**, SOME, ONE, N**ONE**, T**WO**, T**HR**EE. In addition, **SOME** was used as the ambiguous test stimulus, since it could be interpreted either literally (a match) or pragmatically (a mismatch).

There were 4 experimental blocks conforming to the structure of a classic oddball design. Two blocks were *match target* blocks in which most stimuli were mismatches and infrequent ones were matches, which were the blocks' targets, and 2 blocks were *mismatch target* blocks in which standards were matches and infrequent mismatches were the targets. Ambiguous-SOME (**SOME**) appeared in both the block types with its status as target or standard depending on the instructions given to the participants at the beginning of each block (see Section 2.3).

The experiment thus conformed to a 2 × 2 factorial design manipulating Block type (*match target* or *mismatch target*) and Instructions (*pragmatic* or *literal interpretation* of *some*, and consequently *target* or *standard* status of *some* in the block). Within each block (*match target*—*pragmatic some, match target*—*literal some, mismatch target*—*pragmatic some* and *mismatch target*—*literal some*) participants saw: 30 control target-ALL, 30 ambiguous-SOME, and 18 filler targets NONE, ONE, TWO, THREE and SOME. A target or an ambiguous-SOME stimulus was preceded by 3, 4, or 5 pseudo-randomly selected standards (312 in total, 52 of each individual type). There was thus 390 stimuli per block, that is, 312 standards, 30 control targets ALL, 30 ambiguous-SOME, and 18 filler targets. In other words, 20% of the stimuli were deviant targets in the two conditions in which ambiguous-SOME was a target, and 12.3% in the two conditions in which ambiguous-SOME was a standard, see Table 2 below.

**Table 2:** **Design of the ERP experiment**.

**Instruction**	***some* literal**		***some* pragmatic**	
**Block type**	**Match target**	**Mismatch target**	**Match target**	**Mismatch target**
Target stimuli	SOME			**SOME**
	**ALL**	A**LL**	**ALL**	A**LL**
	S**OM**E	SOME	S**OM**E	SOME
	NONE	N**O**NE	NONE	N**O**NE
	O**N**E	ONE	O**N**E	ONE
	T**WO**	T**W**O	T**WO**	T**W**O
	T**HRE**E	T**HR**EE	T**HRE**E	T**HR**EE
Standard stimuli		**SOME**	**SOME**	
	A**LL**	**ALL**	A**LL**	**ALL**
	SOME	S**OM**E	SOME	S**OM**E
	N**O**NE	NONE	N**O**NE	NONE
	ONE	O**N**E	ONE	O**N**E
	T**W**O	T**WO**	T**W**O	T**WO**
	T**HR**EE	T**HRE**E	T**HR**EE	T**HRE**E

### 2.3. Procedure

During EEG cap installation, participants rated a random sequence of the 253 statements of the questionnaire. They were instructed to indicate how strongly they agreed or disagreed with each statement by pressing one of 4 buttons on a response box. They were told that there was no right or wrong answer^3^. Participants generally needed 20–30 min to complete the questionnaire, which generally provided enough time to set the EEG cap.

Participants were then instructed to monitor stimuli presented in the center of a 19′′ CRT monitor in Arial Narrow size 14 points subtending around 1° of visual angle and to press a button if the stimulus differed from the most common type presented within a block. For instance in the *match target*—*pragmatic some* block, participants were instructed to press a button for match stimuli (such as **ALL** or O**N**E) and ignore mismatch stimuli (such as A**LL**, SOME, N**ONE**, ONE, T**WO**, T**HR**EE). They were particularly instructed about **SOME**, which was to be considered a mismatch (and thus ignored) because “not some, but all letters are in green.” In the *match target*—*literal some* block, instructions were the same as above, with the exception of the particular instruction about **SOME**, which was to be considered a match (and thus a target) because “some letters are indeed green.” The *pragmatic some* and *literal some mismatch target* blocks were a mirror image of the two previously described ones, such that standards were now match stimuli (**ALL**, S**OM**E, NONE, O**N**E, T**WO**, T**HRE**E) and targets were mismatch stimuli (e.g., A**LL**, T**HR**EE). Under pragmatic instruction, **SOME** was thus to be considered a mismatch target and responded to, and under literal instruction it was to be considered a match standard and ignored, hence its instruction-dependent status.

Block types (*match target* or *mismatch target*) order and Instructions (*literal* or *pragmatic* interpretation of *some* first) were rotated between participants (8 combinations). Response side was counterbalanced between participants. There was a break between blocks and a short familiarization with specific instructions at the beginning of each block. Each stimulus was presented for 1300 ms or until participant's response, whichever was the shortest, with a randomly selected inter-stimulus interval of 160, 180, 200, 220, or 240 ms to reduce cross-trial ERP contamination. Participants needed around 40 min to complete the task.

### 2.4. EEG recording and analysis

Electrophysiological data were recorded continuously at a rate of 1 *kHz* in reference to electrode Cz from 64 Ag/AgCl electrodes using SynAmp2 amplifiers (Neuroscan Inc., El Paso, TX, USA). Electrodes were attached to an elastic cap (Easycap™, Herrsching, Germany) and placed according to the extended 10–20 convention. The ground electrode was placed at FPz. Bipolar electrodes were placed to the left of the left eye and to the right of the right eye (HEOG) and above and below the right eye (VEOG). Signals were filtered on-line between 0.01 and 200 *Hz*. Impedances were kept below 5 *kΩ* for the 64 electrodes and below 10 *kΩ* for the eye electrodes. Before segmentation, the EEG was processed through a low-pass filter with a cut-off frequency of 20 *Hz* and a high-pass filter of 0.1 *Hz*. Eye blinks were mathematically corrected based on the procedure advocated by Gratton et al. (1983). After correction, any trial with amplitude exceeding ± 100 μV at any point within an epoch and at any recording site except VEOG and HEOG was discarded from analysis. The EEG was then segmented into epochs ranging from −100 to 1000 *ms* after stimulus onset. Baseline correction was performed in reference to pre-stimulus activity, and individual averages were digitally re-referenced to the global average reference. EEG data processing was done using Scan Edit 4.5 (Neuroscan Inc.). Twelve individual datasets were discarded due to excessive noise and/or alpha contamination leading to undetectable early components (P1–N1 complex) in two or more of the blocks. In the remaining 39 datasets, one block was missing due to a technical error and one block with less than 17 accepted trials was discarded, leading to an average number of trials per condition of 28.6 (*SD* = 2).

We expected a delayed P300 effect because of the nature of the task (see e.g., Fosker and Thierry, 2005; Delplanque et al., 2006; Polich, 2007; Thierry and Kotz, 2008; Otterbein et al., 2012; Wu and Thierry, 2013; Sassenhagen et al., 2014). Differences in the early P300 range (P3a) were not analyzed because no clearly differentiated peak was identified. Inspection of the grand-average ERP waveforms at the predicted electrode location of maximal amplitude (Pz, see e.g., Duncan-Johnson and Kopell, 1981; Polich, 2007; Sassenhagen et al., 2014) revealed that the main peak in the later P300 range (P3b) was delayed by about 100 *ms* in the *mismatch target* as compared to the *match target* blocks (grand-average peak latencies 620 and 515 *ms*, respectively). This delay could be expected considering reaction times differences between blocks (see Section 3.2.2). P3b mean amplitudes were computed and analyzed in 150 ms-wide windows around the average peak latency calculated in match and mismatch block types separately: 440–590 *ms* in *match target* blocks and 550–700 *ms* in *mismatch target* blocks, based on visual inspection of variations of the Mean Global Field Power measured across the scalp (Picton et al., 2000; Luck, 2005). P3b mean amplitudes were measured at electrode locations Pz, POz, PO3, PO4.

## 3. Results

### 3.1. Pragmatism score

Out of a maximum of 30, Pragmatism scores of the 39 participants kept for statistical analyses of ERP results ranged from 0 to 29 (*M* = 5.48, *SD* = 8.35). Pragmatism scores did not allow us to split the participants into two groups (pragmatic vs. literal responders) because 8 of them scored the median value of 1.

### 3.2. Behavioral results

#### 3.2.1. Accuracy

**Figure 1:**
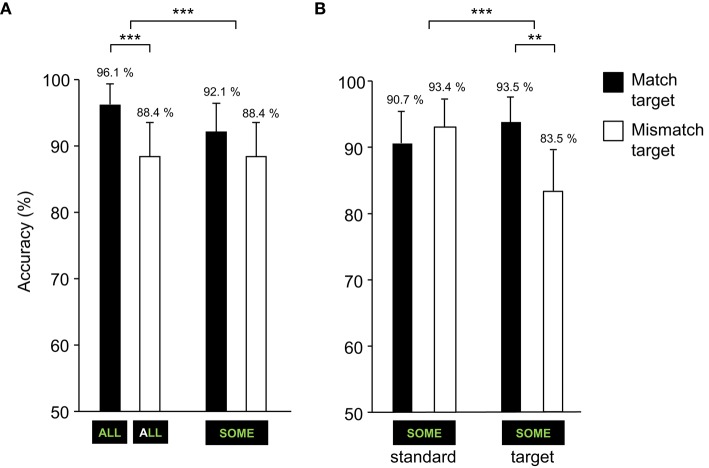
**Correct responses to target-ALL and ambiguous-SOME depending on the *match* or *mismatch target* Block type (error bars represent SEM)**. **(A)** Correct responses to target-ALL and ambiguous-SOME. **(B)** Correct responses to ambiguous-SOME depending on its status in the block (*standard* or *target*). ^***^p < 0.001, ^**^p < 0.01, ^*^p < 0.05.

Hit rates were high overall (91.3 %, *SD* = 28.2). The proportions of correct responses per block types (*match target* and *mismatch target*) and stimulus conditions (target-ALL and ambiguous-SOME, the latter could be considered either a target or a standard depending on the blocks' instructions) are presented in Figure 1.

Hit rates were analyzed using logit mixed models^4^ (see e.g., Jaeger, 2008) including the maximal random effect structure justified by the design and by model comparison^5^, namely by-subject random intercepts and by-subject random slopes for Block type for all models.

The first model revealed a significant Block type × Stimulus interaction (*z* = 5.24, *p* < 0.001), see Figure 1A. Analyses for the stimuli separately showed a significantly higher accuracy in *match* relative to *mismatch target* blocks for target-ALL (*z* = 5.48, *p* < 0.001), see Figure 1A. For ambiguous-SOME, there was a significant interaction Block type × Status (target or standard in the block) (*z* = −7.47, *p* < 0.001), see Figure 1B.

When SOME was intended as a target (*literal* interpretation in *match target* block, and *pragmatic* interpretation in *mismatch target* block), participants made more errors in the *mismatch* than *match target* block (*z* = −3.1, *p* < 0.01), see Figure 1B, there was no interaction with Pragmatism score. When SOME was intended as a standard, and thus was to be ignored (*pragmatic* interpretation in *match target* block, and *literal* interpretation in *mismatch target* block), there was no significant difference between *mismatch* and *match target* blocks (*z* = 1.36, *p* = 0.18), see Figure 1B, nor any interaction with Pragmatism score.

#### 3.2.2. Reaction times

**Figure 2:**
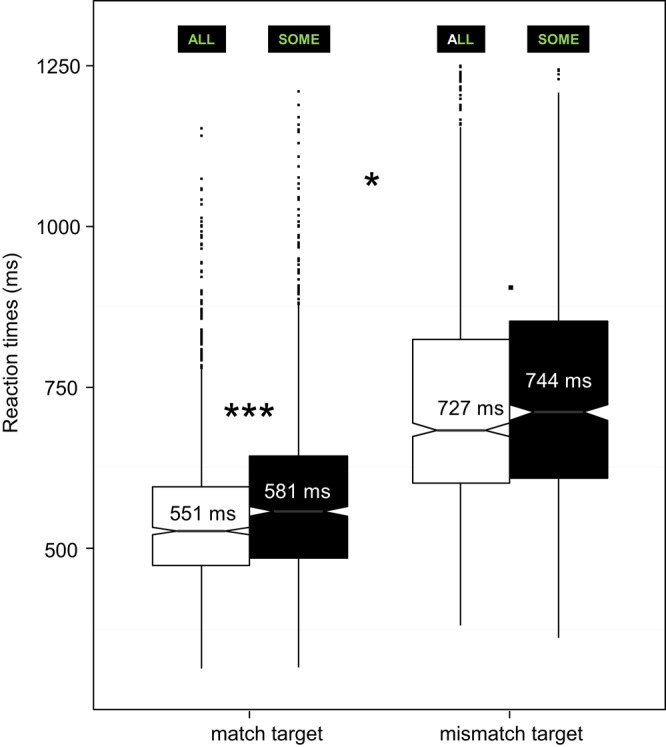
**Reaction times to target-ALL and target-SOME depending on the *match* and *mismatch target* Block type**. Notches give a roughly 95% confidence interval for comparing medians (see e.g., McGill et al., 1978). Integers in the boxes indicate means. ^***^p < 0.001, ^**^p < 0.01, ^*^p < 0.05, ^▪^*p* < 0.1.

This analysis only concerns reaction times for the stimuli ALL and SOME to which participants had to respond, that is target-ALL and target-SOME, in the blocks in which both were targets. Figure 2 depicts reaction times (in ms) per Block type and Stimulus.

Reaction times were analyzed using linear mixed models^6^ (see e.g, Bates, 2005; Baayen et al., 2008; Baayen and Milin, 2010) including maximal random structure justified by the design and supported by the data: by-subject random intercepts and by-subject random slopes for Block type × Stimulus (or for Block type or for Stimulus). Reaction times were transformed according to the Box-Cox power transformation^7^: 1/sqrt(RT). The final models included removal of outliers (data points with absolute standardized residuals exceeding 2.5 standard deviations, see e.g, Baayen and Milin, 2010).

The first model showed a significant interaction between Block type and Stimulus [*F*_(1, 37.17)_ = 4.16, *p* < 0.05]^8^, see Figure 2. Separate analyses for the target stimuli showed a significant effect of Block type for target-ALL [*F*_(1, 34.18)_ = 276.67, *p* < 0.001]. This effect was found for target-SOME too [*F*_(1, 34.36)_ = 165.77, *p* < 0.001], however there was no interaction with Pragmatism score, even though such interaction could have been expected for this stimulus. The effect of Block type on reaction times for both target-ALL and target-SOME corroborates the facilitation effect of *match target* blocks observed on hit rates.

Analyses for the Block types separately showed a significant effect of target Stimulus in the *match target* block [*F*_(1, 36.27)_ = 24, *p* < 0.001]. However, this effect was only marginal in the *mismatch target* block [*F*_(1, 34.93)_ = 3.4, *p* = 0.07]. There was no interaction with Pragmatism score, even though such an interaction could have been expected here also.

In sum, mismatch target detection led to longer reaction times than match target detection (by about 170 *ms*). Furthermore, regardless of tolerance to pragmatic violations as indexed by Pragmatism score, when participants were instructed to take *some* in its literal interpretation (*match target* block), they needed more time to respond to target-SOME than to target-ALL (by about 30 *ms*), but the difference between target-ALL and target-SOME was smaller (about 18 *ms*) when they had to consider target-SOME in its pragmatic interpretation (*mismatch target* block). This suggests that the facilitation effect of the literal interpretation of target-SOME compared with its pragmatic interpretation observed on hit rates only reflects the general facilitation effect of the Block type (*match target* compared with *mismatch target*). Furthermore, the increase of accuracy accompanied by the slowdown of response speed (when comparing target-SOME with the control target-ALL) in the *match target* block resembles a speed-accuracy trade-off.

### 3.3. ERP results (P3b)

**Figure 3:**
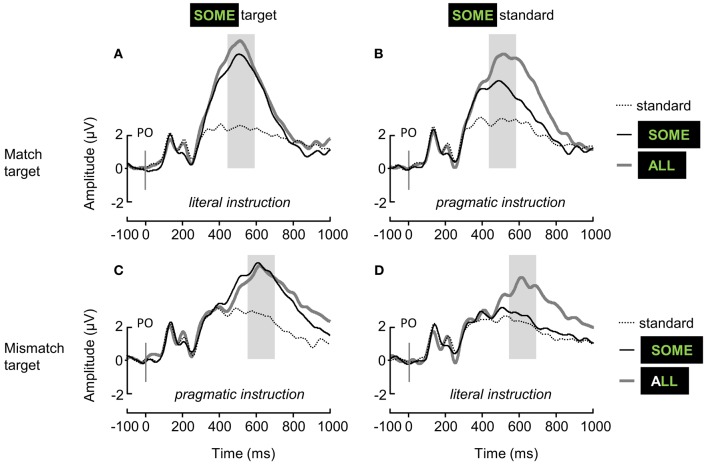
**Grand-average ERP waveforms elicited over the Parieto-Occipital region (linear derivation of Pz, PO3, PO4, POz) by standard items (dotted gray lines), unambiguous target-ALL (solid gray lines), and ambiguous-SOME (solid black lines), depending on the Block type (A,B, *match target*; C,D, *mismatch target*), and the status of ambiguous-SOME (A,C, *target*; B,D, *standard*)**. In italics: the context of interpretation of ambiguous-SOME. The shaded areas represent the time-windows used for the analysis of P3b mean amplitudes.

Grand-average ERP waveforms are displayed in Figure 3. Grand-average difference ERP waveforms and topographies of the P3b effect elicited by target-ALL and ambiguous-SOME are displayed in **Figures 5**, **6**, respectively.

P3b mean amplitudes and P3b mean effects were analyzed using linear mixed models including maximal crossed random effects justified by the data (see Barr et al., 2013, and Sections 3.2.1, 3.2.2). The random structures were kept maximal for all of the models, that is by-subject random intercepts with by-subject random slopes for all of the fixed effects, and by-electrode random intercepts, but for the first model of analysis of the P3b effects for which the complex random structure had to be simplified, see below. The final models included removal of outliers (data points with absolute standardized residuals exceeding 2.5 standard deviations, see Section 3.2.2).

We first analyzed the P3b mean amplitudes elicited by the 3 different Stimulus types (standard, target-ALL and ambiguous-SOME) in the 4 experimental blocks, see Figure 3. We conducted the analyses by block because of the dual status of ambiguous-SOME (*target* or *standard*, see left and right panels in Figure 3), and because the standards were different in the different Block types (*mismatch* or *match target*, see upper and lower panels in Figure 3).

**Figure 4:**
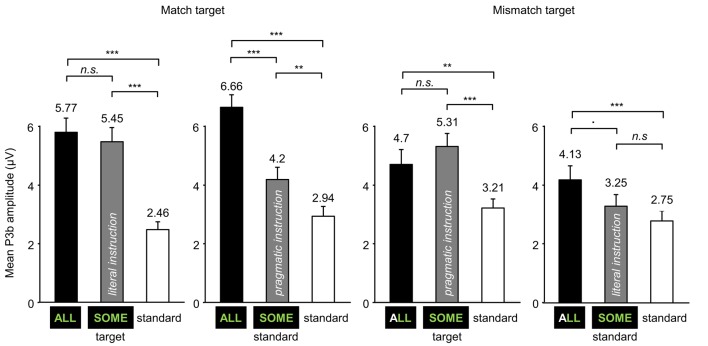
**Mean P3b amplitudes elicited over the Parieto-Occipital region (Pz, PO3, PO4, POz) by ALL, SOME, and standards in each of the four experimental blocks (error bars represent standard errors of the mean)**. ^***^p < 0.001, ^**^p < 0.01, ^*^p < 0.05, ^▪^*p* < 0.1.

In all four blocks, the effect of Stimulus type was significant (*match target* block with *literal* target-SOME, see Figure 3A: *F*_(2, 38.13)_ = 30.71, *p* < 0.001; *match target* block with *pragmatic* standard-SOME, see Figure 3B: *F*_(2, 38.04)_ = 60.87, *p* < 0.001; *mismatch target* block with *pragmatic* target-SOME, see Figure 3C: *F*_(2, 37.04)_ = 28.16, *p* < 0.001; *mismatch target* block with *literal* standard-SOME, see Figure 3D: *F*_(2, 37.07)_ = 10.88, *p* < 0.001); differences between stimuli with estimates of the mixed models in μV and *p*-values adjusted are presented in Table 3, and mean P3b amplitudes in Figure 4).

**Table 3:** **Differences in P3b mean amplitudes**.

**Block type**	**Estimate (SE)**	***p***
**MATCH TARGET**			
Standard vs. target-ALL	3.27 (0.42)	< 0.001
Standard vs. target-SOME	2.94 (0.43)	< 0.001
Target-ALL vs. target-SOME	−0.33 (0.27)	n.s
Standard vs. target-ALL	3.71 (0.34)	< 0.001
Standard vs. standard-SOME	1.26 (0.39)	< 0.01
Standard-SOME vs. target-ALL	2.45 (0.39)	< 0.001
**MISMATCH TARGET**			
Standard vs. target-ALL	1.42 (0.36)	< 0.01
Standard vs. target-SOME	2.04 (0.27)	< 0.001
Target-ALL vs. target-SOME	0.63 (0.34)	n.s
Standard vs. target-ALL	1.36 (0.30)	< 0.001
Standard vs. standard-SOME	0.52 (0.25)	n.s.
Standard-SOME vs. target-ALL	0.84 (0.35)	< 0.1

Estimates (in μV) and p-values adjusted (Bonferroni's correction).

As expected, in all four blocks, mean P3b amplitudes increased for targets as compared with standards. In the blocks in which both ALL and SOME were targets (Figures 3A,C), there was no significant difference between the mean P3b amplitudes elicited by these two stimuli. In the blocks in which only ALL was a target (Figures 3B,D), there was no difference between standard-SOME and the other standards in the *mismatch target* block; but standard-SOME elicited significantly larger P3b amplitudes than other standards in the *match target* block (Figure 3B). Moreover, there was a significant difference between P3b amplitudes elicited by standard-SOME and target-ALL in the *match target* block (Figure 3B), but only a marginal one in the *mismatch target* block (Figure 3D).

In sum, our oddball paradigm delivered the expected effects, but standard-SOME in the *match target* block elicited high P3b amplitude (Figure 3B). In this block, the targets were matches and SOME was to be considered pragmatically, therefore a mismatch, and thus be ignored. Participants managed to ignore the pragmatically mismatched version of SOME very well (accuracy: 90.7%, see Section 3.2.1) but ERPs look as if they had been elicited by a target. However, the difference between standard-SOME and target-ALL in the *mismatch target* block was also less than expected (Figure 3D). In this block in which the only target was ALL, SOME had to be considered literally, so a match and thus had to be ignored. The participants managed to ignore standard-SOME very well (accuracy 93.4%), but although corresponding P3b amplitudes were not significantly different from those elicited by the other standards, they only marginally differed from those elicited by target-ALL. We further discuss these results below. Recall that standard-SOME was rarer than the other standards in the blocks (see Section 2.2.2).

**Figure 5:**
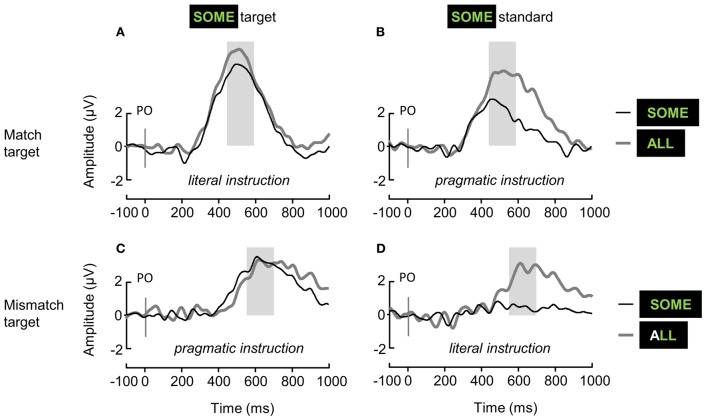
**Grand-average difference ERP waveforms elicited over the Parieto-Occipital region (linear derivation of Pz, PO3, PO4, POz) by unambiguous target-ALL (gray lines), and ambiguous-SOME (black lines), depending on the Block type (A,B, *match target*; C,D, *mismatch target*), and the status of ambiguous-SOME (A,C, *target*; B,D, *standard*)**. In italics: the context of interpretation of ambiguous-SOME. The shaded areas represent the time-windows used for the analysis of P3b effects.

**Figure 6:**
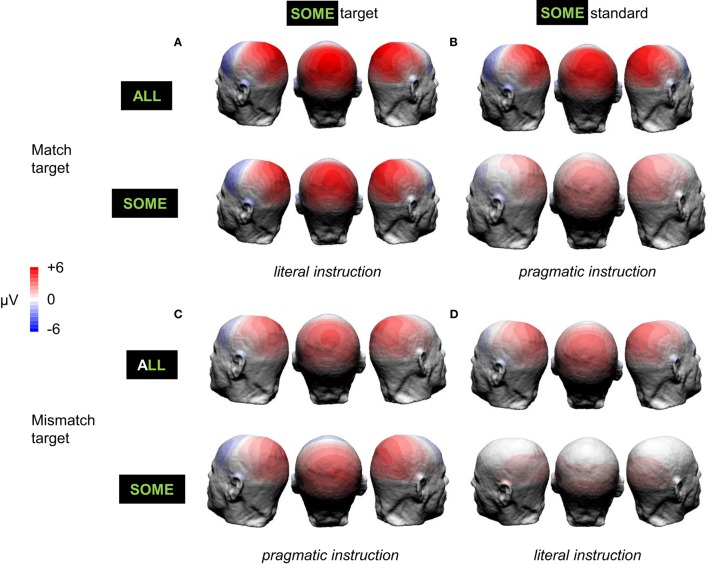
**P3b effect topographies elicited by target-ALL and ambiguous-SOME in *match target* (A,B, 440–590 ms) and *mismatch target* (C,D, 550–700 ms) blocks. (A,C)** ambiguous-SOME as target; **(B,D)** ambiguous-SOME as standard. In italics: the context of interpretation of ambiguous-SOME.

In order to investigate the effect of the interpretation of ambiguous-SOME (*literal* or *pragmatic*), we calculated P3b effects: target-ALL P3b minus standards P3b, and ambiguous-SOME P3b minus standards P3b; see Figure 5 for grand-average difference ERP waveforms and Figure 6 for P3b effect topographies.

The first regression model revealed a significant 4-way interaction between Block type (*match* or *mismatch target*), Status of SOME (target or standard in the experimental block), Stimulus (target-ALL or ambiguous-SOME) and Pragmatism score [*F*_(1, 1039.86)_^9^ = 6.97, *p* < 0.01].

Analyses for target-ALL and ambiguous-SOME separately showed a significant effect of Block type for target-ALL [*F*_(1, 37.85)_ = 25.56, *p* < 0.001] and no effect of, or interaction with, the status of SOME in the block or Pragmatism score. In sum, the P3b effect elicited by target-ALL was reduced in *mismatch target* blocks, regardless of Pragmatism score, and regardless of the status of SOME.

As regards ambiguous-SOME, the first model showed the expected 3-way interaction between Block type (*match* or *mismatch target*), Status of SOME (*target* or *standard* in the block) and Pragmatism score [*F*_(1, 39.4)_ = 5.67, *p* < 0.05]. There was also a significant effect of Block Type [*F*_(1, 36.96)_ = 7.72, *p* < 0.01] and a significant effect of Status [*F*_(1, 37.03)_ = 20.2, *p* < 0.001], but no significant interaction between the two [*F*_(1, 37.45)_ = 2.18, *p* = 0.15]. These effects, and the absence of interaction between them, suggest that whereas standard-SOME elicited the expected reduced P3b effects as compared with target-SOME, it was not processed as a typical standard (it was rarer than the other standards) in any of the blocks. Furthermore, it must be noted that **SOME** was a target in other blocks, it was the only stimulus highlighted by special instructions and was thus task-relevant stimulus even when it was a standard and required no response. The difference that one can see on the figures between standard-SOME in the *match* (Figures 5B, 6B) and the *mismatch target* blocks (Figures 5D, 6D) is similar to that found for target-SOME and target-ALL when comparing across blocks. In other words, this effect is probably one of Block type rather than an effect of the interpretation of SOME, see below.

Analyses for standard-SOME and target-SOME separately showed, for standard-SOME, only a marginal effect of Block type [*F*_(1, 37.83)_ = 3.46, *p* = 0.07]. The P3b effect elicited by standard-SOME decreased by only 0.78 μV (model estimate) in the *mismatch target* block (SOME standard match in its literal interpretation, Figures 5D, 6D) compared with the *match target* block (SOME standard mismatch in its pragmatic interpretation, Figures 5B, 6B). We expected here a possible interaction with Pragmatism score but found none.

**Figure 7:**
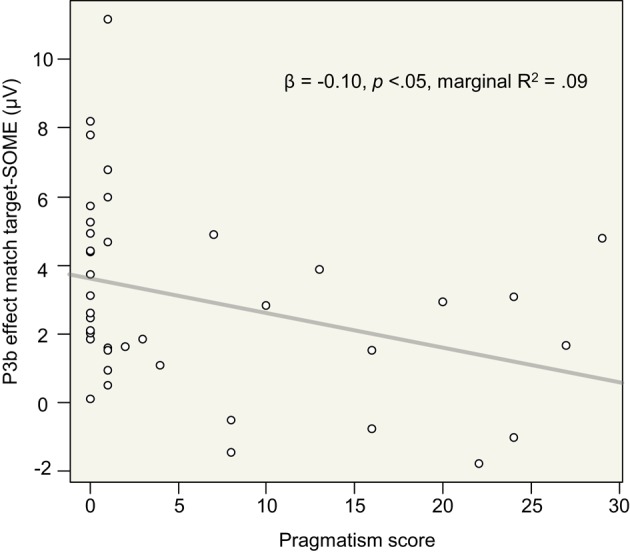
**Mean P3b effect for target-SOME in the match target block (literal instruction) as a function of Pragmatism score in the Parieto-Occipital region (Pz, PO3, PO4, POz)**. R^2^ was calculated using the function *sem.model.fits* of the R package *piecewiseSEM* (Lefcheck, 2016).

As regards target-SOME, analyses revealed the expected significant interaction between Block type and Pragmatism score [*F*_(1, 36.88)_ = 6.72, *p* < 0.05]. Analyses for the Block types separately showed a significant effect of Pragmatism score on P3b effect elicited by target-SOME in the *match target* block [*literal* interpretation of *some*, Figures 5A, 6A, *F*(1, 37) = 4.55, *p* < 0.05], but no effect of Pragmatism score on P3b effect elicited by target-SOME in the *mismatch target* block (*pragmatic* interpretation of *some*, Figures 5C, 6C, *p* > 0.3). In sum, the expected effect of Pragmatism score was only measurable when participants had to take target-SOME in its literal interpretation (*match target* block, Figures 5A, 6A). In this condition, the P3b effect elicited by target-SOME decreased with an increase in Pragmatism score, see Figure 7.

### 3.4. Questionnaire

**Figure A2:**
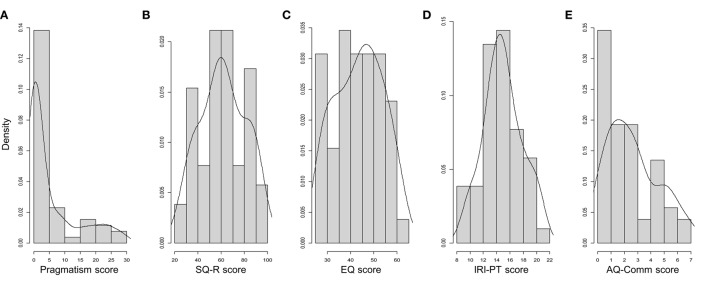
**Distribution of score for each of the measures derived from the questionnaire (see Sections 2.2.1 and 3.4)**. **(A)** Pragmatism score (Mean = 5.48, Median = 1, *SD* = 8.35, Skewness = 1.46.). **(B)** SQ-R score (Mean = 61.58, Median = 61.5, *SD* = 19.63, Skewness = 0). **(C)** EQ score (Mean = 43.6, Median = 44.5, *SD* = 10.32, Skewness = −0.06). **(D)** IRI-Perspective Taking score (Mean = 14.96, Median = 15, *SD* = 2.87, Skewness = 0.14). **(E)** AQ-Communication score (Mean = 2.63, Median = 2, *SD* = 1.95, Skewness = 0.56).

We looked at the results of the 52 participants who completed the questionnaire and included in the analysis the four independent variables of interest: SQ-R score, EQ score, IRI-PT score (*perspective-taking* sub-scale) and AQ-Comm score (*communication* sub-scale)^10^. A generalized linear model (with inverse Gaussian/Wald distribution (link function 1/μ^2^) as exponential family) revealed that SQ-R score was the only significant predictor of Pragmatism score [*t*_(52)_ = −2.206, *p* = 0.032; see in Appendix C, Figure A2 for the distribution, mean, median, SD and skewness values of the dependent and independent variables derived from the questionnaire, and Table A2 for estimates and model comparison]. Pragmatism score tended to increase with an increase in SQ-R score. Systemizing Quotient-Revised score significantly predicted some of the variance in Pragmatism score, whilst the other parameters failed to predict any part of it^11^.

## 4. Discussion

### 4.1. Behavioral responses

Participants made fewer errors when target-SOME was to be taken in its literal (*at-least-some*) rather than its pragmatic (*some-but-not-all*) interpretation. This result is consistent with the pervasive literal interpretation facilitation effect in sentence verification tasks (see e.g., Noveck and Posada, 2003; Bott and Noveck, 2004; De Neys and Schaeken, 2007; Chevallier et al., 2008; Bott et al., 2012). However, in our design, when ambiguous-SOME was a target, interpretation (*literal* or *pragmatic*) and block context (*match* or *mismatch target*) were confounded, and the *mismatch target* experimental context led to generally more errors, that is also for unambiguous target-ALL. In other words, it was easier to detect match targets amidst mismatch standards than the reverse.

Turning to reaction times, when participants were instructed to take *some* in its literal interpretation (*match target* block), they needed more time to respond to target-SOME than to target-ALL, but the difference between the two target types was smaller when they had to consider target-SOME in its pragmatic interpretation (*mismatch target* block). This confirms that the literal facilitation effect observed on hit rates reflects a general facilitation effect of experimental context (Block type). Furthermore, taken together, the increase in hit rates and the slowdown in response speed for literal target-SOME resembles a speed-accuracy trade-off.

We consider here that the literal interpretation facilitation effect found previously in sentence verification tasks might be a general effect of context: it is easier to respond to a “true”/matching than a “false”/mismatching stimulus, even when the response required is the same (as in experiment 2 in Bott and Noveck, 2004, see below). In our experiment, the ambiguous stimulus SOME was always the same: it appeared with all its letters in green. However, it required a response in the *match target* block because it was a match and in the *mismatch target* block because it was a mismatch. In sentence verification tasks, participants have to respond e.g., “true” or “false” to *Some elephants are mammals*, or “agree” to *Mary says the following sentence is* true/false *Some elephants are mammals* (Bott and Noveck, 2004). In any case, **SOME** presented in isolation and statements such as *Some elephants are mammals* are under-informative, and some studies pointed out that the infelicity of under-informativeness probably never goes unnoticed (Feeney et al., 2004; Antoniou and Katsos, 2011, see Section 1). When interpretation is constrained by instructions, responding pragmatically, that is dealing with “false”/mismatching items, appears a harder task than responding literally, that is dealing with “true”/matching items. When interpretation is not constrained, participants can opt for the easy or the harder task based on extraneous variables. In other words, we suggest here that the observed cognitive cost of the pragmatic interpretation of *some* in sentence verification tasks may not entirely originate in deriving the scalar inference *per se* (Bott and Noveck, 2004; Bott et al., 2012) but also in the particular task involved (see also Marty and Chemla, 2013).

### 4.2. P3b brain responses

The P3b is a late peaking positive wave from the P300 family of components (see e.g., Donchin, 1981; Polich, 2007). Its amplitude tends to increase with the propensity of a stimulus to disrupt a sequence of repetitive or ordered events within a predictable sequence. The P3b is expected to be of maximum amplitude in response to stimuli that are most target-like, especially if a response is to be produced by the participant, when stimuli and/or task complexity requires extended processing beyond mere perceptual processing and categorization.

P3b brain responses recorded for the control target stimulus *all* corroborated the effect of experimental context observed on hit rates and response times. It was easier to detect match items amidst mismatch items than the reverse. For the target *some*, there was an interaction between task specific demands and Pragmatism score. Brain responses to *some* literal in the *match target* context decreased with an increase in Pragmatism score. In other words, the more intolerant to pragmatic violations the participant, the weaker the P3b response to literal target *some*. As regards the pragmatic interpretation of *some* in the *mismatch target* block, no effect of Pragmatism score on P3b effect elicited by the target was measurable. As for the case of *some* when it was a standard, we found no interaction with Pragmatism score.

Brain responses corroborated behavioral results: it is more difficult to detect mismatches amid matches than matches amid mismatches, from a semantic or a pragmatic point of view. We found no tangible evidence of cost or delay associated with scalar inference computation (having to infer “not all” from *some*) *per se* when controlling for specific task demands. In this sense, our results are inconsistent with a two-step context-driven model (literal meaning first and optional SI enrichment) as experimental pragmatics has it. Tomlinson et al. (2013) found that when verifying under-informative sentences such as “Some elephants are mammals,” average mouse paths initially moved toward “true” before they changed direction to select “false.” They concluded that SIs are understood in two steps: literal and then pragmatic. However, it is difficult to understand why they invoke such two-step processing model only for “Some elephants are mammals” and not for “No elephants are insects” which produces a comparable response delay. The task seems equally difficult in both cases: there are two consistent linguistic-semantic cues but the response to produce is inconsistent with them (see Urbach and Kutas, 2010; Urbach et al., 2015, for ERP evidence of partial incremental interpretation of quantifiers; and Clark and Chase, 1972, on the processing of “double negative”). Let's imagine a simplified incremental algorithm behind a sentence verification task. For instance, in the case of “Some elephants are mammals”: *some* (exist) *elephants are mammals* (exist), intended response is “false.” For “No elephants are insects”: *no* (¬exist) *elephants are insects* (¬exist), intended response is “true.” Thus, the observed delay may be due to the fact that the response intended has been counter-primed twice. And indeed, this never happened in the other control sentences in Tomlinson et al. (2013). Arguably, judging “No elephants are insects” as “true” is not a pragmatic response because it corresponds to the truth value or logical value, of the sentence. But, it could also be argued that the spontaneous interpretation of “No elephants are insects” is “false.” The double negation elimination may be a valid rule of classical logic (the so-called rule of replacement or *inference*, related to the principle of non-contradiction) but it is not systematically applied, as in the case of the non-standard but frequent double negative in English (e.g., *I didn't say nothing*) which resolves to a negative. In sum, whatever the position one adopts, it is difficult to see why the processing of “Some elephants are mammals” (“false”) would be less “automatic” than the processing of “No elephants are insects” (“true”). The “automatic” computational process appears nonetheless to be more than a one-stage process in sentence verification tasks: it involves (i) accessing the quantifier's value, (ii) computing the semantics of the embedded proposition, (iii) computing the relationship between the quantifier and the embedded proposition (with a literal output for the *some*-sentence and a wrong/contradictory output for the *no*-sentence), and (iv) evaluating the truth value of the sentence in the world. The third stage is easy to complete (exist + exist = true, or ¬exist + ¬exist = false), but the output of fourth stage must be the reverse of the previous stage in order to comply with world knowledge. This might explain why children tend to respond *true* to under-informative statements such as “Some elephants are mammals” more often than adults, and why adults under cognitive load (De Neys and Schaeken, 2007; Marty and Chemla, 2013) or time pressure (Bott and Noveck, 2004; Chevallier et al., 2008) do the same: they are making errors. More generally, the underlying process could be something like “if there is a mismatch or a contradiction resolve it,” and what is important is the value of the mismatch or contradiction to resolve. It could explain why we can encourage adults to be more “logical,” and children to be more “pragmatic” (see e.g., Noveck, 2001). It could also explain why a child so spontaneously says that Charlotte who has eaten *all* of the sweets is a liar when she says that she has eaten *some* of them (see Feeney et al., 2004): the brain is more interested in this than in verifying “Some elephants are mammals” because the former has some value. In this sense, the process is also “context-driven.” Recall that certain specific semantic contexts such as antecedents of conditionals seem to block the “not all” interpretation of *some*, and that in contexts in which the speaker is assumed to have insufficient knowledge of the situation, the hearer does not necessarily access the “not all” interpretation (see Section 1).

The relationship between P3b amplitude and Pragmatism score provided insights into inter-individual variability. Along with a higher Pragmatism score, ambiguous-SOME (**SOME**) was less evident as a match target. This result suggests that P3b amplitude is a sensitive measure of cognitive flexibility and task adaptation. Participants generally managed to switch extremely well from one experimental block to another (*match* or *mismatch* target and *literal* or *pragmatic* interpretation of *some*). However, the relationship trend between intolerance to pragmatic violations and the reduction in the P3b effect elicited by literal *some* suggests that the pragmatic mismatch was less easy to suppress in order to treat *some* literally for some participants.

Alongside the discussion of our results, we have considered circumstantial evidence from other studies. Further investigation is required to characterize the nature of mismatch resolution processes we have hypothesized. Nevertheless, further research in experimental pragmatics should not only consider the principled difficulty of deriving scalar inferences but also that of dealing with mismatches in general (see also Shetreet et al., 2014).

### 4.3. Evaluating intolerance to pragmatic violations based on sentence verification

In the questionnaire, we used under-informative statements such as:

(7) Some infants are young.

in order to evaluate individual intolerance to pragmatic violations.

Although adults tend to be more intolerant to pragmatic violations generally, we found a relative proportion of participants who always, or almost always, strongly agreed with the under-informative statements (Pragmatism score of 0 or 1, 30 participants out of 52). This could be due to the fact that some of the statements we used were similar to (7), which is under-informative because all infants, by definition, are young, but others were like:

(8) Some hammers have a handle.

for which counter-examples or exceptions to the alternative *all*-statement can more easily be found (e.g., old or broken hammers could lack a handle, see also Guasti et al., 2005, pp. 690–691). In such cases, it can be argued that an informative alternative statement would be *Most hammers have a handle* rather than *All hammers have a handle*, which may render (8) more acceptable than (7). Indeed, when Feeney et al. (2004, experiment 3) used only statements of the sort of (8), half of their adult participants gave literal responses only. Guasti et al. (2005, pp. 690–691) argue that such statements can encourage participants to attempt figuring out exceptions to universal statements (e.g., *All hammers have a handle*) in order to make the under-informative statements more sensible and informative. However, Antoniou and Katsos (2011, experiment 2) who controlled the context provided to their participants (who judged, e.g., “There are suns on some of the cards” whilst looking at cards all featuring a sun) found that approximately half of the participants always gave literal responses. Therefore, the fact that the context of evaluation was not controlled in the case of under-informative statements such as (8) probably fails to explain alone why adults are sometimes unexpectedly tolerant to pragmatic violations. Yet participants could have resorted to another strategy leading to the observed preponderance of literal responses and a lack of variation in response types despite our use of a four-level rating scale rather than a binary forced-choice (true/false): the formal settings of the experiment might have invited participants to consider the *some*-statements as a test of logic.

In sum, even when controlling context of evaluation, and despite offering multiple possible choices rather than binary choices, participants appear to develop strategies idiosyncratic to the testing context. In any case, some participants seem to have opted for the easy task (dealing with true/matching rather than false/mismatching statements, see Section 4.1 and 4.2). If we are on the right track with this interpretation of the “agree”/“true”/logical/literal response mode in sentence verification tasks, it is in fact very pragmatic in a broad sense (for computational and human cognition saving principles, see e.g., Montague, 2007).

### 4.4. Pragmatism and systemizing

The score the participants obtained in the Systemizing Quotient-Revised questionnaire was the only significant parameter in the analysis of Pragmatism score. We discuss here some implications of this novel finding although the relationship between personality and cognitive traits and SI derivation requires further investigation.

Apart from “default models,” pragmatic theories assume that SI derivation requires some sort of mind-reading since the hearer has to reason about speaker's knowledge and what she did not say (see Section 1). It could be considered surprising that we found no relationship between Empathy Quotient or Interpersonal Reactivity Index *perspective-taking* sub-scale and Pragmatism score. However, no information about the person producing the statements or other contextual information was provided to the participants and it was thus impossible to work out the producer's intentions or the context in which the statements were made. As regards Autism-Spectrum Quotient *communication* sub-scale and Pragmatism score, the tentative prediction made by Nieuwland et al. (2010, p. 343): “one possible prediction is that high AQ-Comm people are also more likely to respond ‘true’ to under-informative statements in a sentence-verification paradigm” was not supported (see also e.g., Heyman and Schaeken, 2015).

Our data suggest that there may be a relationship between systemizing and intolerance to pragmatic violations, such that Pragmatism score would tend to increase with SQ-R score. This could be seen as an inconsistent result if SQ-R is considered a proxy for logical reasoning. But this could in fact be expected if systemizing is taken to index participants' ability to work out the make up of the experiment and thus their ability to distinguish those statements that are under-informative [e.g., (7) or (8)] from others that are not, e.g.,:

(9) Some birds live in cages.

However, the experimental context alone probably fails to account for our results because the questionnaire featured only 5 true and felicitous *some*-statements, and because *some*- and *all*-statements were intermixed with 213 other statements from the AQ, EQ, SQ-R, and IRI. Another explanation could be that the better the participants at systemizing, the more salient the lexical scale 〈all, some〉 and thus the easier the first step of SI derivation. As suggested by van Tiel et al. (2016, pp. 32–33), hearers might rely on statistical regularities such as: if the speaker uses “some…,” then she means “not all…,” in order to derive SIs. According to Baron-Cohen (2008, p. 66), systemizing leads to identification of rules of the following form: “If X (operation) occurs, A (input) changes to B,” and thus a strong sensitivity to patterns. In other words, it is possible that the better the participants at systemizing, the greater the likelihood of *some* meaning *not all*.

The trend for a positive relationship between intolerance to pragmatic violation and systemizing skills also makes sense in light of the literature on high-functioning autism and Asperger's syndrome. Individuals with such cognitive style are assumed to experience difficulties with pragmatics, however they are as intolerant to pragmatic violations as controls (whether they are adults, Pijnacker et al., 2009; or children, Chevallier et al., 2010). Since they are usually very good at systemizing whilst scoring low on EQ and high on AQ (see e.g., Wheelwright et al., 2006), systemizing skills must help in sentence verification tasks. If we are on the right track with our interpretation of the “agree”/“true”/logical/literal response mode in sentence verification tasks as finally the pragmatic one (in a broad sense: save energy whenever possible), it is no longer expected from individuals with high-functioning autism or Asperger's syndrome to particularly opt for this response mode. Furthermore, since systemizing is linked with attention to detail and leads to the seeking of exact truth (Baron-Cohen, 2008, 2011), it makes sense that participants with high systemizing skills tend to agree less with statements that do not describe reality with high accuracy, that are not optimal.

## 5. Conclusion

Using a novel oddball paradigm with single words and recording hit rates, reaction times and brain activity whilst controlling for task demands, and collecting a measure of inter-individual variation, we failed to replicate a straightforward literal interpretation facilitation effect. Crucially, we provided some evidence to explain why this effect may not be entirely construed as some models of experimental pragmatics have it. We suggest that scalar inference derivation also involves generic, possibly unconscious, albeit cognitively costly and context-driven, procedures for mismatch processing. We argue that the true “pragmatic,” that is efficient, response to under-informative *some*-statements in sentence verification tasks is not “false”/“disagree”/rejection but “true”/“agree”/acceptance: it saves brain energy when not much is at stake.

Overall, we take the view that our data reveal a little more how flexible and adaptive the human cognitive system is.

## Author contributions

Conception, design, and writing: CB and GT. Data collection and statistics: CB.

## Funding

This research was funded by the Swiss National Science Foundation (grant P2NEP1_155426, CB).

### Conflict of interest statement

The authors declare that the research was conducted in the absence of any commercial or financial relationships that could be construed as a potential conflict of interest.

## Footnotes

^1^Note that in fact the response pattern does not always appear bimodal and that some participants can be wholly inconsistent within an experiment. However, criteria for grouping participants vary from one study to another, and inter-individual variation is not always reported.

^2^We followed the same procedure as in Noveck (2001) and Feeney et al. (2004) : by switching the quantifier, true universals of list one became test existentials in list two, and test existentials of list one became true universal in list two; 5 false universals of list one the 5 true existentials in list two, and the 5 true existentials of list one 5 false universals in list two.

^3^The instructions were “Please read each statement carefully and rate how strongly you agree or disagree with it by pressing the corresponding button. There are no right or wrong answers. Please answer as spontaneously as possible.”

^4^Logit mixed models fitted using the R (R Core Team, 2014) package *lme4* (Bates et al., 2014).

^5^See e.g., Barr et al. (2013).

^6^Linear mixed models fitted using the R package *lmerTest* (Kuznetsova et al., 2014).

^7^We used the *boxcox* function of the R package *MASS* (see Venables and Ripley, 2002) to determine the suitable transformation.

^8^We used the *anova* function of *lmerTest* which provides analysis of variance tables of type 3 with denominator degrees of freedom calculated based on Satterthwaite's approximation.

^9^The random structure had to be simplified for this model and therefore does not include the 3-way interaction Block type × Status of SOME × Stimulus as by-subject random slope but only the Block type and the Status of SOME × Stimulus interaction. This simplification was determined based on the *rand* function of *lmerTest* (Kuznetsova et al., 2014).

^10^We verified that there was no problem of multicollinearity calculating the variance inflation factors using the *vif* function of the R package *car* (Fox and Weisberg, 2011).

^11^Fitting linear models with transformed Pragmatism score (1/Pragmatism score^2^) which satisfy all of the linear model assumptions as assessed by the function *gvlma* of the R package *gvlma* (Pena and Slate, 2014) (whereas linear models with untransformed Pragmatism score led to comparable results but violated the normality of the error distribution assumption), we obtained similar results. R^2^ final linear model (SQ-R only predictor): 0.11. $\eta_{p}^{2}$ initial linear model: SQ-R: 0.13, EQ: 0.001, PT: 0.03, AQ-Comm: 0.001.

## Appendix A

**Table A1:** ***All*- and *some*-statements used in the questionnaire (see Section 2.2.1)**.

**Condition**	**Statement**	
	**List 1**	**List 2**
True universals	All bikes have wheels	All airplanes have wings
	All books have pages	All cars have engines
	All elephants have trunks	All cats have ears
	All hammers have a handle	All circles are round
	All infants are young	All fish can swim
	All refrigerators have doors	All freezers are cold
	All robins have wings	All gangs have members
	All rock bands have musicians	All giraffes have long necks
	All sentences have words	All people have lungs
	All zebras have stripes	All televisions have screens
False universals	All animals are black	All children are blonde
	All birds live in cages	All dresses have pockets
	All books have color pictures	All drinks are made from chocolate
	All clothes have zippers	All flowers are yellow
	All dogs have spots	All tools are made of wood
	All birds have telephones	All birds have telephones
	All chairs tell the time	All chairs tell the time
	All couches have windows	All couches have windows
	All crayons have noses	All crayons have noses
	All garages sing	All garages sing
True existentials	Some children are blonde	Some books have color pictures
	Some dresses have pockets	Some clothes have zippers
	Some drinks are made from chocolate	Some dogs have spots
	Some flowers are yellow	Some animals are black
	Some tools are made of wood	Some birds live in cages
False existentials	Some books are good to eat	Some books are good to eat
	Some children are made of feathers	Some children are made of feathers
	Some fruits have computers	Some fruits have computers
	Some mammals are made of leaves	Some mammals are made of leaves
	Some stores are made of bubbles	Some stores are made of bubbles
Test existentials	Some airplanes have wings	Some bikes have wheels
	Some cars have engines	Some books have pages
	Some cats have ears	Some elephants have trunks
	Some circles are round	Some hammers have a handle
	Some fish can swim	Some infants are young
	Some freezers are cold	Some refrigerators have doors
	Some gangs have members	Some robins have wings
	Some giraffes have long necks	Some rock bands have musicians
	Some people have lungs	Some sentences have words
	Some televisions have screens	Some zebras have stripes

## Appendix C

**Table A2:** **Model comparison (see Section 3.4)**.

	**Null model**	**Initial model**	**Final model**
Intercept	0.00417 (0.00062)^***^	0.00520 (0.00434)	0.00863 (0.00224)^***^
SQ-R		−0.00007 (0.00003)^*^	−0.00007 (0.00003)^*^
EQ		−0.00001 (0.00007)	
IRI-PT		0.00026 (0.00023)	
AQ-Comm		0.00006 (0.00035)	
AIC	337.41	336.43	332.64
BIC	341.31	348.13	338.49
Log Likelihood	−166.70	−162.21	−163.32
Num. obs.	52	52	52

Estimates and standard errors (in brackets).

*** p < 0.001,

^**^p < 0.01,

* p < 0.05.

An inverse Gaussian distribution was used as family, negative estimates should be read as positive estimates, and positive estimates as negative ones. Table realized using the R package texreg (Leifeld, 2013).
